# Correlates of anti-EBV EBNA1 IgA positivity among unaffected relatives from nasopharyngeal carcinoma multiplex families

**DOI:** 10.1038/bjc.2011.502

**Published:** 2011-11-17

**Authors:** C M Chang, K J Yu, W L Hsu, J M Major, J Y Chen, P J Lou, M Y Liu, S R Diehl, A M Goldstein, C J Chen, A Hildesheim

**Affiliations:** 1 Division of Cancer Epidemiology and Genetics, National Cancer Institute, NIH, DHHS, 6120 Executive Blvd., EPS 7073, Rockville, MD, USA; 2 Graduate Institute of Epidemiology, College of Public Health, National Taiwan University, Taipei, Taiwan; 3 Genomics Research Center, Academia Sinica, Taipei, Taiwan; 4 National Institute of Cancer Research, National Health Research Institutes, 35, Keyan Road, Zhunan Town, Miaoli County 350, Taiwan; 5 Department of Otolaryngology, National Taiwan University Hospital and National Taiwan University College of Medicine, Taipei, Taiwan; 6 Center of General Education, National Taipei University of Nursing and Health Sciences, Taipei, Taiwan; 7 Center for Pharmacogenomics and Complex Disease Research, New Jersey Dental School, University of Medicine and Dentistry of New Jersey, Newark, NJ, USA

**Keywords:** Epstein–Barr virus, EBNA1, VCA, IgA, DNase, nasopharyngeal carcinoma

## Abstract

**Background:**

To determine whether non-viral nasopharyngeal carcinoma (NPC) risk factors might be associated with (and mediated through) Epstein–Barr virus (EBV) serological responses linked to NPC risk, we evaluated predictors of risk of anti-EBNA1 IgA seropositivity and other markers among unaffected relatives from a large NPC family study in Taiwan.

**Methods:**

Multivariate logistic regression conditioned on family was used to examine the associations between sociodemographic, dietary, lifestyle, and occupational variables and risk of anti-EBV EBNA1 IgA positivity, anti-VCA IgA, and anti-DNase positivity.

**Results:**

Among 2393 unaffected relatives from 319 multiplex families, 1180 (49.3%) were anti-EBV EBNA1 IgA seropositive. None of the associations with anti-EBNA1 IgA were statistically significant, except for being 31–50 years of age (*vs* <30, adjusted ORs 0.51–0.57). For one or more EBV serological markers, there were suggestive associations for older age, GuangDong firm salted fish, betel use, current alcohol use, and male gender.

**Conclusion:**

Overall, we found little evidence to suggest that non-viral NPC risk factors significantly alter EBV serological patterns, suggesting that non-viral NPC risk factors act through pathways independent of EBV serological responses.

Nasopharyngeal carcinoma (NPC) is rare, albeit relatively common in southern China, southeast Asia, the Arctic, and North Africa (Chang and Adami, 2006). NPC risk factors include male gender, increased age, southern Chinese ethnicity, less education, consumption of salted fish and other preserved foods containing elevated levels of nitrate/nitrosamines, reduced fruit and vegetable consumption, cigarette smoking, betel nut chewing, alcohol consumption, and occupational exposures (Chang and Adami, 2006). Epstein–Barr virus (EBV), a ubiquitous herpesvirus that infects over 90% of adults worldwide, is believed to be necessary, but not sufficient for developing NPC (Chang and Adami, 2006). Longitudinal studies in China and Taiwan have shown that positivity for anti-EBNA1 IgA, anti-VCA IgA, and anti-DNase antibodies were associated with a significantly elevated NPC risk (Zeng *et al*, 1983, 1985; Chien *et al*, 2001; Ji *et al*, 2007; Hsu *et al*, 2009; Ng *et al*, 2010; Yu *et al*, 2011).

Whether non-viral risk factors act independently of anti-EBV serological responses or mediate EBV effects on NPC development is not known. To address this question, we evaluated the association between non-viral NPC risk factors and EBV serological responses in a group of healthy individuals from high-risk NPC multiplex families in Taiwan.

## Methods

Participants in the present study were part of the NPC multiplex family study in Taiwan, previously described (Pickard *et al*, 2004; Yang *et al*, 2005; Yu *et al*, 2009). Over 300 NPC multiplex families were identified and recruited, including 659 NPC cases and 2557 unaffected parents, siblings, spouses, and children.

Risk factors were assessed by questionnaire for all individuals by a trained nurse. Sociodemographic characteristics were age, gender, ethnicity, and education. Lifestyle factors included smoking, betel use, and alcohol use. Occupational exposures evaluated included duration of formaldehyde exposure and wood exposure.

Dietary intake during ages 10–30 (representing consumption during adolescence and adulthood) was assessed by food frequency questionnaire, such as: consumption of salted fish, other preserved foods (salted meat, smoked foods, preserved eggs, fried/fermented bean curd, fermented rice, and fermented flour sauce), and fruits and vegetables. Other preserved foods were combined into a single variable by summing across the variables, and dividing into categories corresponding to the questionnaire diet frequency categories.

Serum from participants was tested for the following EBV antibody markers: anti-VCA IgA by the immunofluorescent assay, anti-EBNA1 IgA by enzyme-linked immunosorbent assay, and anti-DNase by an enzyme neutralisation assay as previously described (Pickard *et al*, 2004). For the present analysis, a total of 2393 unaffected family members from 319 NPC multiplex families with data on EBV serology were included.

All estimates in this analysis were obtained using conditional logistic regression with the SAS PHREG procedure (SAS version 9.2), conditioning on families to account for familial correlations (Pfeiffer *et al*, 2003). We chose anti-EBNA1 IgA (at a cut-off of OD_405_ ⩾0.10) as the primary outcome of interest for this analysis, because this measure was optimised for high sensitivity and most strongly predicted NPC risk in our family study (Yu *et al*, 2011). In secondary analyses, we evaluated (1) anti-EBNA1 IgA (OD_405_⩾0.20) with an alternative cut-off used for studies of sporadic NPC, (2) anti-VCA IgA based on the cut-off of 1 : 10 or greater dilution of serum, and (3) DNase neutralising activity, based on a cut-off optimised for high sensitivity in our family study (positivity cut-off=160 or more neutralising units; Yu *et al*, 2011). We also evaluated anti-EBNA1 IgA without the more ambiguous group (0.10⩽OD_405_<0.20), so that a clean, positive group (OD_405_⩾0.20) was compared with a clean, negative group (OD_405_<0.10). However, the results resembled those of the original outcome (OD_405_⩾0.20 *vs* OD_405_<0.20), and therefore, were not presented.

Variables selected for consideration in the adjusted models were based on significant or borderline significant associations (based on the 95% confidence intervals (CI)) with EBNA1 positivity (OD_405_⩾0.10). Dose response was based on calculating overall Wald *P*-values for categorical variables.

## Results

There were a total of 2393 unaffected family members in this study. The mean age was 46 years (47 in women and 44 in men), and 53% were women.

Overall, there were 1180 (49.3%) individuals seropositive for anti-EBNA1 IgA (OD_405_⩾0.10). In the full model, being 31–40 years old and 41–50 years old (*vs* 18–30 years) were inversely associated with anti-EBNA1 IgA positivity (adjusted OR (aOR)=0.51, 95% CI=0.32–0.83 and aOR=0.57, 95% CI=0.35–0.91, respectively), but associations with the older age groups were not significant. There was a suggestive, nonsignificant association between GuangDong firm salted fish (*vs* never; aOR=1.8) and anti-EBNA1 positivity (Table 1).

A total of 384 (16.1%) individuals were seropositive for anti-EBNA1 IgA (OD_405_⩾0.20). Current alcohol use (*vs* never use) was associated with anti-EBNA1 IgA positivity (aOR=1.7, 95% CI=1.0–2.8; Table 1). Former betel use (*vs* never use; aOR=1.9) and GuangDong firm salted fish, and both mouldy and firm salted fish (*vs* never; aORs 1.6–1.9) were nonsignificantly associated with anti-EBNA1 IgA positivity. There were nonsignificantly inverse associations with anti-EBNA1 IgA positivity for the three middle age groups (31–40, 41–50, and 51–60 years; aORs 0.63–0.86), but no association with the oldest age group (Table 1).

A total of 491 (26.3%) individuals were seropositive for anti-VCA IgA. Compared with the youngest age group (less than 30), the oldest age group (greater than 60; aOR=1.9, 95% CI=1.1–3.5) was associated with anti-VCA IgA positivity (*P*-trend=0.012). Not reaching statistical significance, GuangDong firm salted fish (*vs* never; aOR=2.0, 95% CI=0.88–4.5) was associated with anti-VCA IgA positivity (Table 1).

A total of 767 (32.1%) individuals were seropositive for anti-DNase. Former and current betel use (*vs* never use) and GuangDong firm salted fish (*vs* never) were significantly associated with anti-DNase seropositivity (aORs 2.2–2.9). Males were at lowered risk of anti-DNase seropositivity compared with females (aOR=0.64, 95% CI=0.43–0.94). Age greater than 30 (*vs* less than 30) was associated with anti-DNase seropositivity (aORs 1.8–2.5, *P*-trend=0.81).

## Discussion

With a couple of notable exceptions discussed below, we saw little evidence to indicate that non-viral NPC risk factors influence anti-EBV seroreactivity. This suggests that non-viral NPC risk factors are unlikely to influence NPC risk by altering anti-EBV serological profiles.

We observed a U-shaped curve for age, with anti-EBNA1 IgA positivity being higher among the youngest and oldest, and lower among the middle age categories. The higher antibody positivity rate observed among older individuals, also seen for anti-VCA IgA, could reflect immunosenescence in older age, leading to more frequent viral lytic reactivation (Agarwal and Busse, 2010). The fact that anti-EBNA1 IgA positivity, but not other markers, was also higher among the youngsters, is not clearly understood.

There was a suggestive elevated risk for all EBV markers (aORs 1.6–2.7) associated with GuangDong firm salted fish, but not mouldy fragrant fish. In an *in vitro* study, aqueous extracts of Cantonese salted fish activated EBV lytic replication in Raji cells in a dose-dependent manner by causing cells to express EBV early antigen (Shao *et al*, 1988). However, it is unclear why one type of GuangDong salted fish would activate EBV, but not another type.

A significant association was observed between betel use and anti-DNase positivity, and was suggestive for anti-EBNA1. Although betel nut use is classified as a group 1 carcinogen in humans, there is no data on whether or not betel use can lead to EBV reactivation (IARC, 2004). Betel nut ingredients have induced inflammation *in vitro*, supporting the biological plausibility of this association (Jeng *et al*, 2000, 2003; Chang *et al*, 2005).

There may be recall bias of diet between the ages of 10–30, such that young subjects may be prone to recall their diet in adolescence and older subjects prone to recall diet in their adulthood. EBV serology was measured at only one point in time and may not capture all episodes of EBV lytic replication. We would not have detected associations with other aspects of EBV exposure and/or host response to EBV. Our results may have been affected by the reproducibility for anti-VCA IgA testing, which was modest (agreement∼68%, *κ*∼0.29–0.38; Pickard *et al*, 2004). Our findings for a high-risk population may not represent that of the general population, and associations may be attenuated due to similarity of exposures within high-risk families (Yang *et al*, 2005). However, by studying unaffected relatives from NPC multiplex families, the population is enriched in terms of EBV IgA antibody positivity, which occurs at much lower frequencies in the general population (Pickard *et al*, 2004). Additional strengths of the study include the large sample size, recruitment through cases identified from the national cancer registry, availability of detailed risk factor information, as well as multiple EBV serological markers.

In summary, the majority of NPC risk factors were not found to be significantly associated with anti-EBNA1 IgA positivity, the strongest predictor of NPC risk in our study population. In conclusion, our data suggest that non-viral NPC risk factors affect NPC risk via mechanisms other than through effects on EBV reactivation or host antibody responses to such infections.

## Figures and Tables

**Table 1:** Adjusted odds ratios (aORs)^a^ for the associations between NPC risk factors and risk of EBV antibody seropositivity for anti-EBNA IgA (OD_405_⩾0.1), anti-EBNA IgA (OD_405_⩾0.2), anti-VCA IgA (⩾1:10), and anti-DNase (⩾160)

	**Anti-EBNA1 IgA-positive (⩾0.1)**				**Anti-EBNA1 IgA-positive (⩾0.2)**				**Anti-VCA IgA-positive (⩾1:10)**				**Anti-DNase-positive (⩾160)**			
**Factor**	**Total**	***N***	**%**	**aOR (95% CI)**	**Total**	***N***	**%**	**aOR (95% CI)**	**Total**	***N***	**%**	**aOR (95% CI)**	**Total**	***N***	**%**	**aOR (95% CI)**
*Age*																
18–30	429	216	50.4	1.0 (Reference)	429	64	14.9	1.0 (Reference)	307	66	21.5	1.0 (Reference)	429	99	23.1	1.0 (Reference)
31–40	558	250	44.8	0.51 (0.32, 0.83)	558	74	13.3	0.63 (0.34, 1.2)	424	97	22.9	1.1 (0.66, 1.9)	558	194	34.8	2.5 (1.5, 4.3)
41–50	546	268	49.1	0.57 (0.35, 0.91)	546	90	16.5	0.70 (0.38, 1.3)	429	112	26.1	1.7 (0.96, 2.8)	546	163	29.9	1.8 (1.1, 3.1)
51–60	372	191	51.3	0.78 (0.48, 1.3)	372	65	17.5	0.86 (0.46, 1.6)	314	86	27.4	1.6 (0.96, 2.8)	372	133	35.8	2.1 (1.2, 3.6)
>60	488	255	52.3	0.77 (0.45, 1.3)	488	91	18.7	1.1 (0.58, 2.2)	390	130	33.3	1.9 (1.1, 3.5)	488	178	36.5	1.8 (1.0, 3.3)
*P*-trend				0.74				0.18				0.012				0.81
																
*Gender*																
Female	1269	588	46.3	1.0 (Reference)	1269	187	14.7	1.0 (Reference)	994	275	27.7	1.0 (Reference)	1269	429	33.8	1.0 (Reference)
Male	1124	592	52.7	1.1 (0.73, 1.5)	1124	197	17.5	1.1 (0.68, 1.7)	870	216	24.8	0.81 (0.54, 1.2)	1124	338	30.1	0.64 (0.43, 0.94)
																
*Smoking*																
Never	1616	762	47.2	1.0 (Reference)	1616	235	14.5	1.0 (Reference)	1260	327	26.0	1.0 (Reference)	1616	514	31.8	1.0 (Reference)
Former	186	105	56.5	1.7 (0.91, 3.0)	186	43	23.1	1.4 (0.71, 2.8)	148	39	26.4	0.98 (0.52, 1.9)	186	59	31.7	0.66 (0.35, 1.3)
Current	585	310	53.0	0.95 (0.63, 1.4)	585	104	17.8	0.97 (0.58, 1.6)	450	123	27.3	1.3 (0.86, 2.1)	585	192	32.8	1.5 (0.97, 2.3)
																
*Betel*																
Never	2143	1033	48.2	1.0 (Reference)	2143	330	15.4	1.0 (Reference)	1669	436	26.1	1.0 (Reference)	2143	675	31.5	1.0 (Reference)
Former	84	52	61.9	1.3 (0.66, 2.8)	84	25	29.8	1.9 (0.84, 4.2)	69	22	31.9	1.0 (0.45, 2.2)	84	31	36.9	2.9 (1.4, 6.1)
Current	159	91	57.2	1.3 (0.72, 2.4)	159	27	17.0	1.1 (0.51, 2.4)	119	31	26.1	0.77 (0.39, 1.5)	159	58	36.5	2.2 (1.2, 4.2)
																
*Alcohol*																
Never	1818	873	48.0	1.0 (Reference)	1818	272	15.0	1.0 (Reference)	1409	374	26.5	1.0 (Reference)	1818	584	32.1	1.0 (Reference)
Former	100	56	56.0	0.8 (0.36, 1.6)	100	23	23.0	0.7 (0.28, 1.8)	81	18	22.2	0.62 (0.28, 1.4)	100	33	33.0	0.68 (0.32, 1.5)
Current	469	248	52.9	1.1 (0.77, 1.7)	469	87	18.6	1.7 (1.0, 2.8)	368	97	26.4	1.1 (0.74, 1.7)	469	148	31.6	1.2 (0.82, 1.9)
																
*Duration of formaldehyde*																
None	1042	484	46.5	1.0 (Reference)	1042	182	17.5	1.0 (Reference)	1042	301	28.9	1.0 (Reference)	1042	382	36.7	1.0 (Reference)
<10 years	155	71	45.8	1.3 (0.84, 2.0)	155	18	11.6	0.78 (0.42, 1.5)	155	36	23.2	0.77 (0.48, 1.3)	155	58	37.4	1.1 (0.71, 1.7)
⩾10 years	216	122	56.5	1.3 (0.84, 2.0)	216	53	24.5	1.4 (0.83, 2.4)	216	81	37.5	1.1 (0.67, 1.7)	216	81	37.5	1.1 (0.67, 1.6)
*P*-trend				0.14				0.25				0.87				0.78
																
*Fruits and vegetables*																
Less than once a day	257	142	55.3	1.0 (Reference)	257	47	18.3	1.0 (Reference)	194	51	26.3	1.0 (Reference)	257	93	36.2	1.0 (Reference)
1–2 times a day	1324	645	48.7	0.88 (0.54, 1.4)	1324	202	15.3	0.98 (0.53, 1.8)	1025	269	26.2	0.86 (0.51, 1.5)	1324	411	31.0	0.69 (0.42, 1.1)
2 or more times a day	784	377	48.1	0.86 (0.51, 1.5)	784	128	16.3	0.86 (0.45, 1.6)	622	162	26.1	0.95 (0.54, 1.7)	784	248	31.6	0.80 (0.47, 1.4)
*P*-trend				0.62				0.46				0.99				0.68
																
*GuangDong salted fish*																
Never	1941	960	49.5	1.0 (Reference)	1941	300	15.5	1.0 (Reference)	1474	410	27.8	1.0 (Reference)	1941	610	31.4	1.0 (Reference)
Mouldy fragrant fish only	45	23	51.1	1.4 (0.58, 3.3)	45	6	13.3	0.78 (0.22, 2.7)	42	10	23.8	1.0 (0.38, 2.7)	45	15	33.3	0.76 (0.31, 1.9)
Firm fish only	60	36	60.0	1.8 (0.86, 3.9)	60	14	23.3	1.6 (0.58, 4.2)	57	18	31.6	2.0 (0.88, 4.5)	60	25	41.7	2.7 (1.1, 6.5)
Both mouldy and firm fish	98	40	40.8	0.98 (0.51, 1.9)	98	24	24.5	1.9 (0.85, 4.3)	90	14	15.6	0.84 (0.40, 1.8)	98	35	35.7	1.3 (0.7, 2.5)

a Adjusted odds ratios are from full models containing the following variables: age, gender, smoking, betel use, alcohol use, duration of formaldehyde exposure, fruit and vegetable intake, and type of salted fish intake.
